# Investigation on Low-temperature Annealing Process of Solution-processed TiO_2_ Electron Transport Layer for Flexible Perovskite Solar Cell

**DOI:** 10.3390/ma13051031

**Published:** 2020-02-25

**Authors:** Xing Yu, Xiaoping Zou, Jin Cheng, Dan Chen, Yujun Yao, Chuangchuang Chang, Baoyu Liu, Junqi Wang, Zixiao Zhou, Guangdong Li

**Affiliations:** 1 Beijing Advanced Innovation Center for Materials Genome Engineering, Research Center for Sensor Technology, Beijing Key Laboratory for Sensor, MOE Key Laboratory for Modern Measurement and Control Technology, School of Applied Sciences, Mechanical and Electrical Engineering School, Beijing Information Science and Technology University, Beijing 100101, China; nimingyx1@163.com (X.Y.); chengjin@bistu.edu.cn (J.C.); yyj10zy@gmail.com (Y.Y.); changcc037@gmail.com (C.C.); liubaoyu0214@163.com (B.L.); 13126706081@163.com (J.W.); zzxfpp111@163.com (Z.Z.); LGD1511455720@163.com (G.L.); 2 State Key Laboratory on Integrated Optoelectronics, Institute of Semiconductors, Chinese Academy of Sciences, Beijing 100083, China; chendan1988@semi.ac.cn; 3 Center of Materials Science and Optoelectronics Engineering, University of Chinese Academy of Sciences, Beijing 100049, China

**Keywords:** perovskite solar cells, flexible, low-temperature annealing process, solution-processed TiO_2_ electron transport layer

## Abstract

Flexible perovskite solar cells (PSCs) have received increasing attention in wearable and portable devices over the past ten years. The low-temperature process of electron transport layer plays a key role in fabricating flexible PSCs. In this paper, we improve the performance of flexible PSCs by controlling the thermodynamic procedure in the low-temperature annealing process of solution-processed TiO_2_ layers and modulating the precursor concentration of (6,6)-phenyl c_61_ butyric acid methyl ester (PC_61_BM) deposited on fluorine-doped tin oxide (FTO)/TiO_2_ substrate. The results show that slowing down evaporation rate of residual solvent and adopting PC_61_BM of appropriate precursor concentration are confirmed to be effective methods to improve the performance of flexible PSCs. We also demonstrate carbon electrode-based flexible PSCs. Our work expands the feasibility of low temperature process for the development of flexible perovskite photodetectors and light-emitting diodes, as well as flexible PSCs.

## 1. Introduction

Organometal halide perovskite solar cells (PSCs) have aroused great interest in academia and industries [1,2,3,4,5]. The environmental issues concerning the footprint of PSC manufacturing processes are also of great importance [6]. Flexible PSCs expand the feasibility for the application in wearable and portable devices [7,8,9,10]. Recently, the power conversion efficiency (PCE) of flexible PSCs has rocketed to 18.4% [10].

There are many papers on low fabrication oxide. For example, Wang et al. adopted solution-processed amorphous WO_X_ as electron selective layer for PSCs [1], and Eze et al. Fabricated efficient planar PSCs using solution-processed amorphous WO_X_/fullerene C_60_ as electron extraction layer [2]. In this paper, we use TiO_2_ as electron transport layer (ETL). However, the traditional TiO_2_ ETL-based PSCs require high temperature sintering (>400 °C) [11,12,13,14,15], which exceeds the thermal budget of flexible substrates such as FTO-coated polyethylene terephthalate (PET) (≤150 °C). Therefore, low temperature process of TiO_2_ ETL has been intensively studied [16,17,18,19,20]. Yang et al. and Feleki et al. demonstrated magnetron sputtering and electron beam to prepare highly dense TiO_2_ layer, respectively [16,17]. A solution process was also reported to fabricate compact TiO_2_ ETL [18].

In 2019, based on solution process, Chu et al. studied the influence of treatment time of UV/O_3_ process following the annealing process of TiO_2_ film on a hotplate on the properties of TiO_2_ ETL and discovered that 80 min-UV/O_3_-treating endowed the flexible PSCs with a best PCE of 7.33% owing to UV/O_3_-induced removal of residual organic solvent as well as enhanced hydrophilicity and conductivity of TiO_2_ films [19]. In the same year, You et al. investigated the effect of different solvent used to re-disperse TiO_2_ nano-sol on the quality of solution-processed TiO_2_ ETL prior to the annealing process of TiO_2_ film on a hotplate and demonstrated flexible PSCs of better PCE of 15.8% based on N, *N*-dimethylformamide (DMF) solvent, which has high zeta potential and low surface tension [20].

In this paper, we prepare the nano TiO_2_ films with solution process and perform the annealing process on a hotplate at low temperature (≤150 °C). We control the morphology of TiO_2_ ETL by adjusting evaporating rate of the organic solvent in the annealing process on a hotplate, which leads to the change of thermodynamic process in the growth and formation of TiO_2_ films. Furthermore, we optimize the ETL with PC_61_BM of different precursor solution to reduce the defects on the surface of TiO_2_ layer [21]. It is worth noting that all the exploring experiments are carried out on the rigid substrates at first, and then we prepare the carbon electrode-based flexible PSCs based on the optimized preparing process.

## 2. Experiments

### 2.1. Materials

FTO (7–8 Ω/square, Hunan Yiyang Xiangchen Tech Co., Ltd., Yiyang, China), low-temperature TiO_2_ nanoparticle (LT-TiO_2_ NP) (Shanghai Mater Win New Materials Co., Ltd., Shanghai, China), PC_61_BM (Xi’an Polyme Light Technology Corp., Xi’an, China), DMF (99.8%, Shanghai Mater Win New Materials Co., Ltd., Shanghai, China), dimethyl sulfoxide (DMSO, 99.8%, Shanghai Mater Win New Materials Co., Ltd., Shanghai, China), methyl ammoniur iodide (CH_3_NH_3_I, >99.5%, white powder in appearance, Shanghai Mater Win New Materials Co., Ltd., Shanghai, China), lead(II) iodide (PbI_2_) (99.99%, yellow crystalline powder in appearance, Xi’an Polyme Light Technology Corp., Xi’an, China), 2, 2′, 7, 7′-tetrakis-(N, N-dip-methoxyphenylamine)-9, 9′-spirobifluorene solution (Spiro-OMeTAD) (≥99.5%, yellow solution in appearance, Xi’an Polyme Light Technology Corp., Xi’an, China).

### 2.2. Device Fabrication

#### 2.2.1. Cleaning of FTO Substrate

First, the FTO conductive glass was cleaned with deionized water (Beijing Information Science and Technology University, Beijing, China) and detergent with ultrasonic vibration cleaners (KQ-100E, Skymen Cleaning Technology Shenzhen Co., Ltd., Shenzhen, China) for 20 min. Next, FTO was cleaned with ethanol (Sinopharm Chemical Reagent Co., Ltd., Beijing, China) for 20 min. Then, FTO was cleaned with a mixed solution of isopropyl alcohol (Sinopharm Chemical Reagent Co., Ltd., Beijing, China), acetone (Sinopharm Chemical Reagent Co., Ltd., Beijing, China) and deionized water in a volume ratio of 1:1:1 for 20 min. Finally, the cleaned conductive glass was treated with UV light cleaner (BZS250GF-TC, Shenzhen Huiwo Technology Co., Ltd., Shenzhen, China) for 15 min.

#### 2.2.2. Formation of Electron Transport Layer

LT-TiO_2_ NP solution was deposited on the FTO substrate by spin-coating at 2000 rpm for 30 s and then dried at 150 °C for 20 min on a hot plate to form a nano TiO_2_ layer.

(1) Giving the heating mode, we determine three kinds of annealing procedures (Process 1): (a) The annealing temperature was directly raised to 150 °C (Direct method); (b) The annealing temperature was raised from room temperature to 150 °C at rate of 8 °C/min (Proportional method); (c) The as-grown nano TiO_2_ layer had been delayed for one hour before the annealing temperature was raised from room temperature to 150 °C at rate of 8 °C/min (Delayed method);

(2) Based on Process 1(c), we demonstrated an interface modifying procedure (Process 2): Depositing PC_61_BM of different precursor concentration (5, 10, 15, 20, 25 mg/mL) on nano TiO_2_ layer by spin-coating at 1500 rpm for 30 s. After the deposition, PC_61_BM was left in the ambient condition for forty minutes to evaporate slowly with no annealing process. It is worth noting that the PC_61_BM was dissolved in chlorobenzene (Shanghai Mater Win New Materials Co., Ltd., Shanghai, China).

#### 2.2.3. Formation of Perovskite Light-absorption Layer

First, the PbI_2_ was fully dissolved in a mixed solvent of DMF and DMSO in the volume ratio of 0.95:0.05 by vigorous stirring to form 600 mg/mL precursor solution. The methyl ammonium was fully dissolved in anhydrous isopropanol to form 70 mg/mL precursor solution. Then, the PbI_2_ precursor solution was deposited on the PC_61_BM layer by spin-coating at 1500 rpm for 30 s. Immediately, the CH_3_NH_3_I precursor solution was drop-cast on the as-grown PbI_2_ film at 1500 rpm for 30 s and then dried at 150 °C for 20 min.

#### 2.2.4. Formation of Hole Transport Layer

The hole transport layer was formed by spin-coating Spiro-OMeTAD on perovskite layer at 3000 rpm for 30 s. After the spin-coating, Spiro-OMeTAD was left in the ambient condition for 40 minutes to evaporate slowly with no annealing process.

#### 2.2.5. Formation of Counter Electrode

The soot from the burning candle was collected with an FTO glass substrate to form a sponge-like carbon electrode, which was then pressed against the prepared hole transport layer [22].

### 2.3. Characterization

Field emission transmission electron microscope (FE-TEM) (Tecnai G2 F20, FEI, Hillsboro, OR, USA), field emission scanning electron microscope (FE-SEM) (SU8020, Hitachi, Tokyo, Japan) images were obtained for structural and morphological characterization of TiO_2_ films, PC_61_BM layers and perovskite films. The current-voltage (J–V) curves were obtained under standard simulated air-mass (AM) 1.5 sunlight generating from a solar simulator (Sol 3A, Oriel, Newport, RI, USA). UV-vis absorption spectra were characterized by ultroviolet visible absorption spectrometer (Avantes, Apeldoom, The Newtherlands). Photoluminescence (PL) spectrum was measured with PL testing system (LabRAW HR800, HORIBA Jobin Yvon, Paris, France). All the characterization of devices was performed in ambient atmosphere at room temperature.

## 3. Results and Discussion

**Figure 1:**
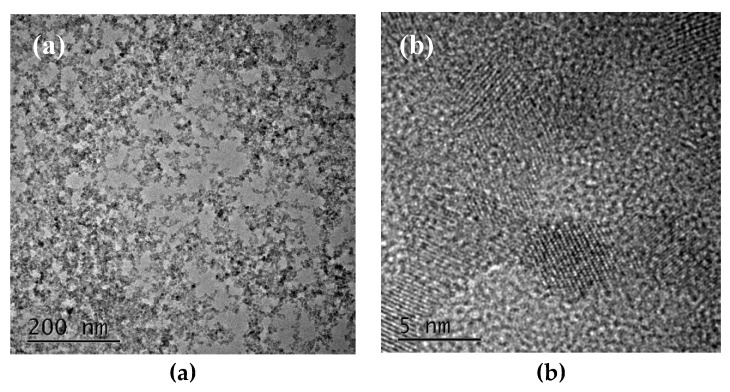
Transmission electron microscope (TEM) images of TiO_2_ nanoparticles: (**a**) The low magnification; (**b**) The high magnification.

In this paper, we investigate the effect of low temperature process of conventional TiO_2_-based ETL on the performance of PSCs. Hence, we have characterized the structure of TiO_2_ nanoparticles with TEM, as demonstrated in Figure 1, showing the average size of about 5 nm.

**Figure 2:**
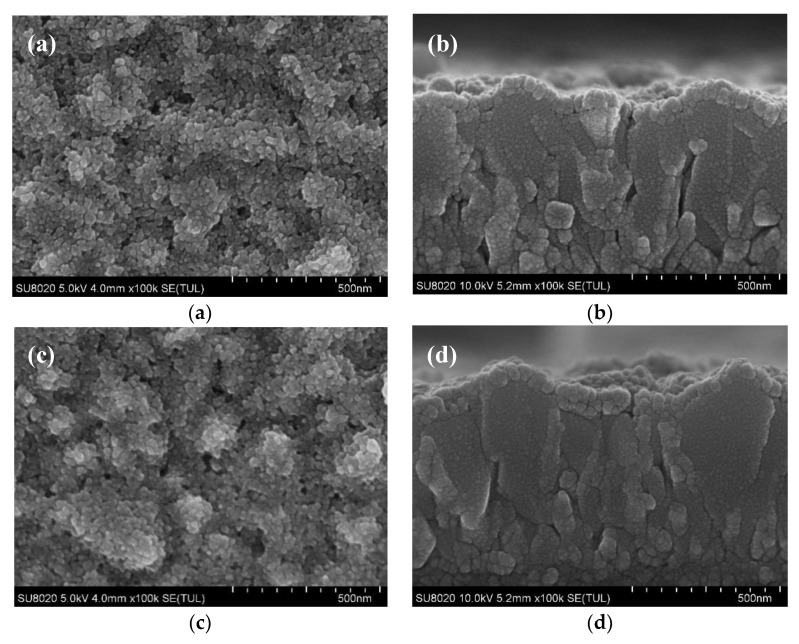

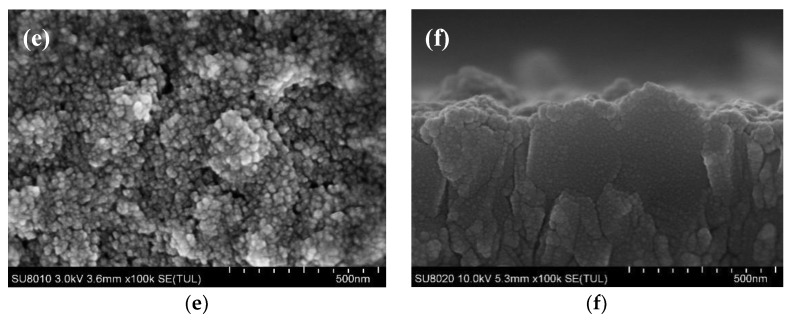
Scanning electron microscope (SEM) images of nano TiO_2_ films using Process 1: (**a**,**c**,**e**) The top-view images under Direct method, Proportional method, and Delayed method, respectively; (**b**,**d**,**f**) The corresponding cross-sectional images. The meaning of Process 1, Direct method, Proportional method and Delayed method is described in the second paragraph of experimental Section 2.2.2.

**Figure 3:**
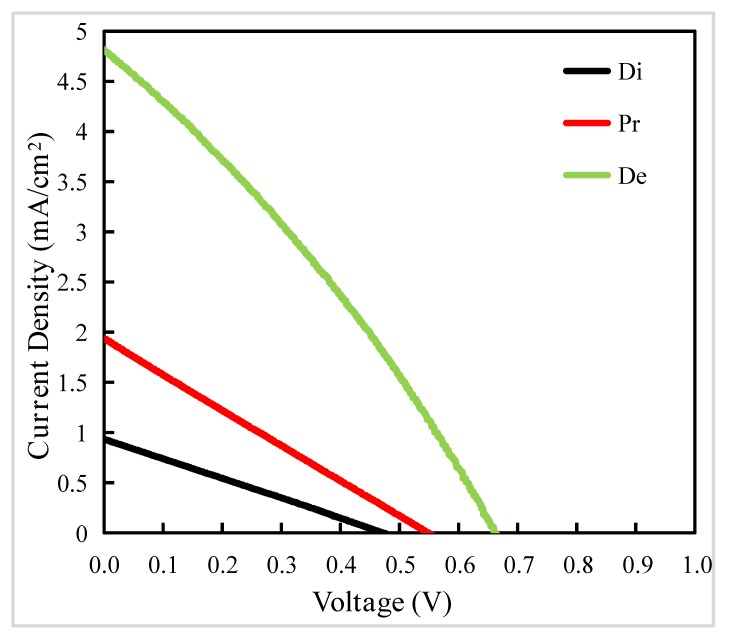
Reverse scanning J–V curves of PSCs based on Process 1. The letter Di, Pr, De represent films prepared with Direct method, Proportional method, and Delayed method, respectively. The device active area is 0.25 cm^2^ (0.5 cm × 0.5 cm). The meaning of Process 1, Direct method, Proportional method and Delayed method is described in the second paragraph of experimental Section 2.2.2.

Firstly, we investigate the influence of thermodynamic process on the growth and formation of nano TiO_2_ films. The detailed information about experiments is shown in the second paragraph of experimental Section 2.2.2. The SEM images are shown in Figure 2. The J–V curves of PSCs and the corresponding data extracted from J–V curves are shown in Figure 3 and Table 1, respectively.

It is clearly observed from Figure 2a that there are large holes remained in nano TiO_2_ film prepared with Direct method. Figure 2b shows that these holes locate at valleys of FTO where the surface curvature changes greatly. Some voids can still be discovered from Figure 2c and d obtained from nano TiO_2_ film prepared under Proportional method, however, they are smaller compared with Figure 2a,b. Figure 2f shows the FTO is effectively covered by nano TiO_2_ film with few defects fabricated under Delayed method. We can also observe that the nano TiO_2_ layer and FTO surface fit well with each other, indicating the conformal growth of nano TiO_2_ on FTO substrate.

We can conclude that the morphology of nano TiO_2_ film deposited on FTO substrate can be controlled by optimizing annealing process and nano TiO_2_ film exhibits high quality when Delayed method was introduced, which is ascribed to the slower evaporating rate of the residual solvent, resulting in conformal covering of TiO_2_ over FTO substrate. Generally, films with fewer cracks and defects endow the PSCs with better performance, which is also confirmed by reverse scanning J–V curves of the devices in Figure 3 and data extracted from reverse scanning J–V curves in Table 1.

**Figure 4:**
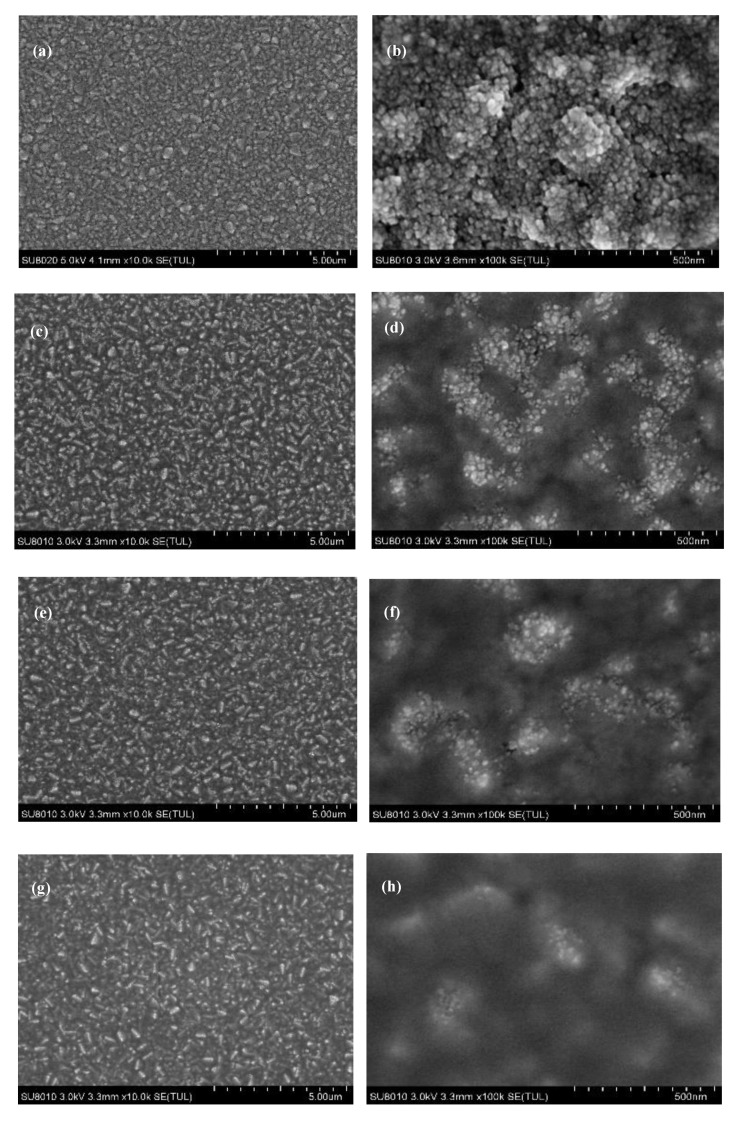

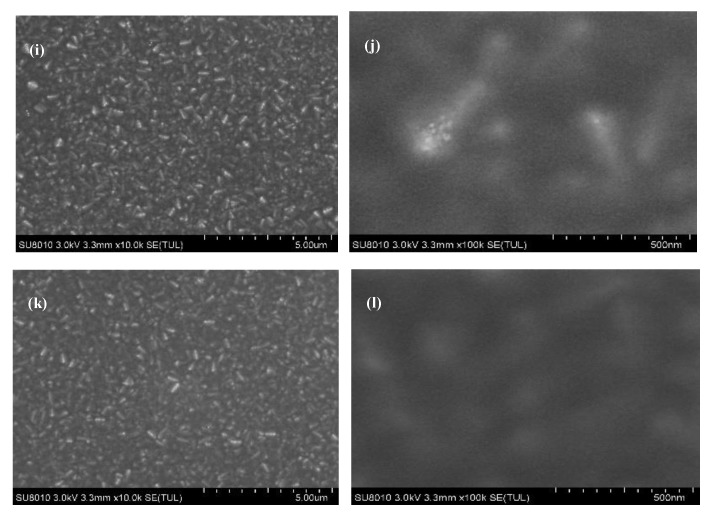
SEM images of nano TiO_2_ layer under Process 2: (**a**,**c**,**e**,**g**,**I**,**k**) Represent 0, 5, 10, 15, 20, 25 mg/mL, respectively, the low magnification; (**b**,**d**,**f**,**h**,**j**,**l**) The corresponding high magnification. The meaning of Process 2 is described in the third paragraph of experimental Section 2.2.2.

Secondly, as shown in Figure 2, the TiO_2_ nanoparticles are not connected tightly with each other, which may lead to leakage of PSCs. Therefore, we studied the effect of PC_61_BM on the interface between nano TiO_2_ layer and perovskite layer. The detailed information about experiments is shown in the third paragraph of experimental Section 2.2.2. The SEM images are shown in Figure 4.

As shown in Figure 4d,f,h,j,l, it is clearly observed that there are some light islands distributed on the dark substrate. We can determine that the light islands are peaks of nano TiO_2_ layer and the dark substrate is PC_61_BM comparing with Figure 4b. From the magnified SEM images, we can conclude that the islands disappear gradually with the concentration of PC_61_BM increasing from 5 to 25 mg/mL. When the concentration of PC_61_BM is 5 mg/mL, the PC_61_BM just filled the valleys of nano TiO_2_ layers. With the concentration of PC_61_BM increased to 15 mg/mL, the nano TiO_2_ film was well covered by PC_61_BM and we can hardly find the light islands, leaving few cracks. While the concentration further rises to 25 mg/mL, the nano TiO_2_ layer was fully covered by PC_61_BM, giving smooth and uniform film.

As demonstrated in previous report [23], it is too hard for thick ETL to transfer the electron from the absorption layer to FTO substrate and instead relatively thin ETL leads to a serious recombination of electrons and holes. Hence, we can infer that the device optimized with PC_61_BM of 15 mg/mL exhibits the best performance, in which PC_61_BM filled the most valleys of nano TiO_2_ layer, leaving few cracks, preventing the contact between FTO substrate and perovskite layer and improving the electron transporting efficiency.

**Figure 5:**
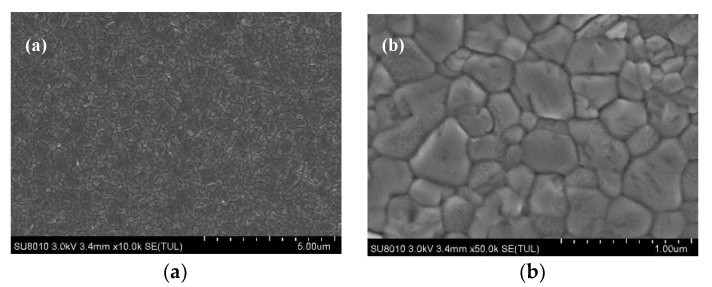

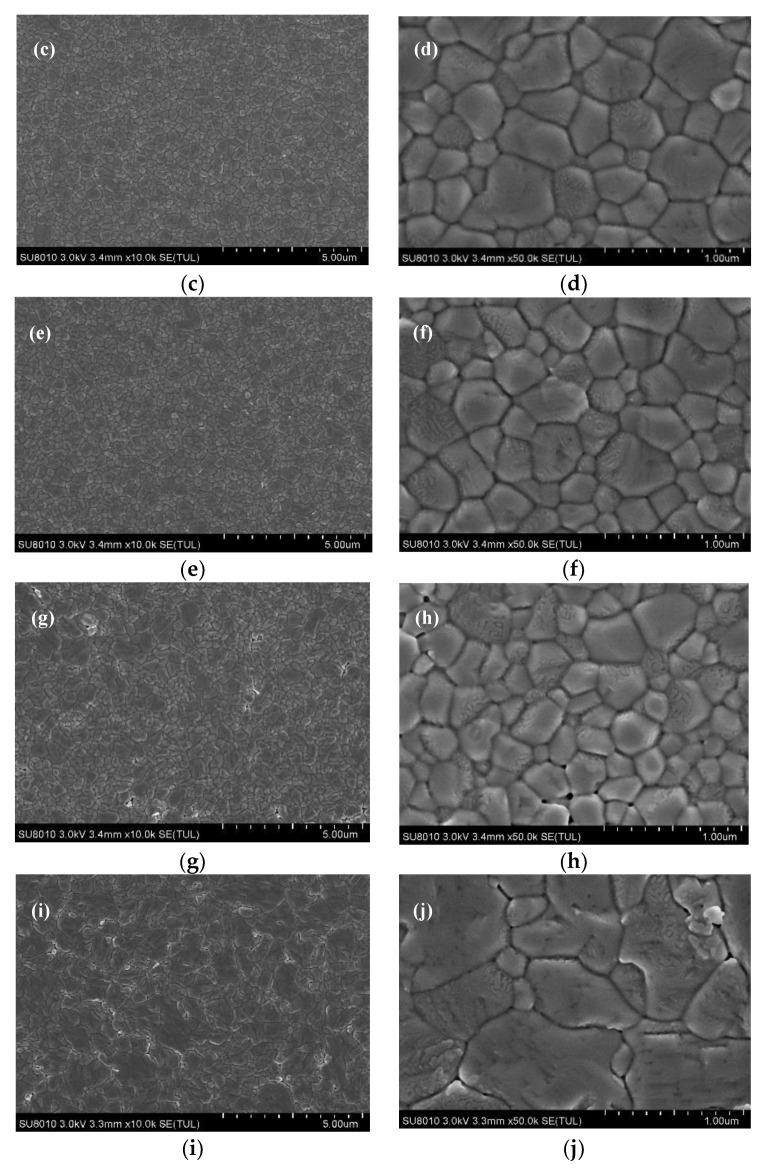
SEM images of perovskite film prepared on nano TiO_2_ optimized with (6,6)-phenyl c_61_ butyric acid methyl ester (PC_61_BM) of different concentration: (**a**,**c**,**e**,**g**,**i**) The low magnification; (**b**,**d**,**f**,**h**,**j**) The high magnification.

**Figure 6:**
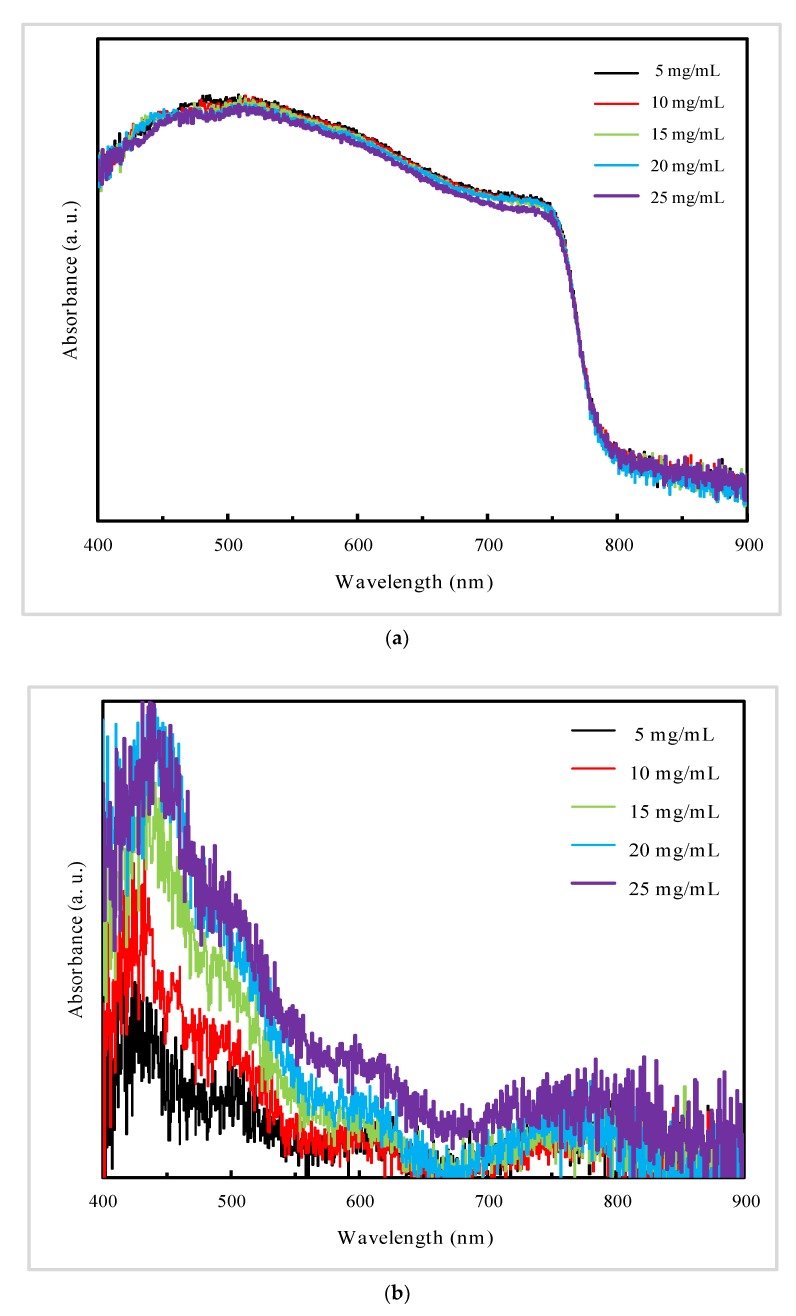
UV–vis absorption spectra of films: (**a**) Perovskite films deposited on PC_61_BM layers of different concentration.; (**b**) PC_61_BM films of different concentration.

**Figure 7:**
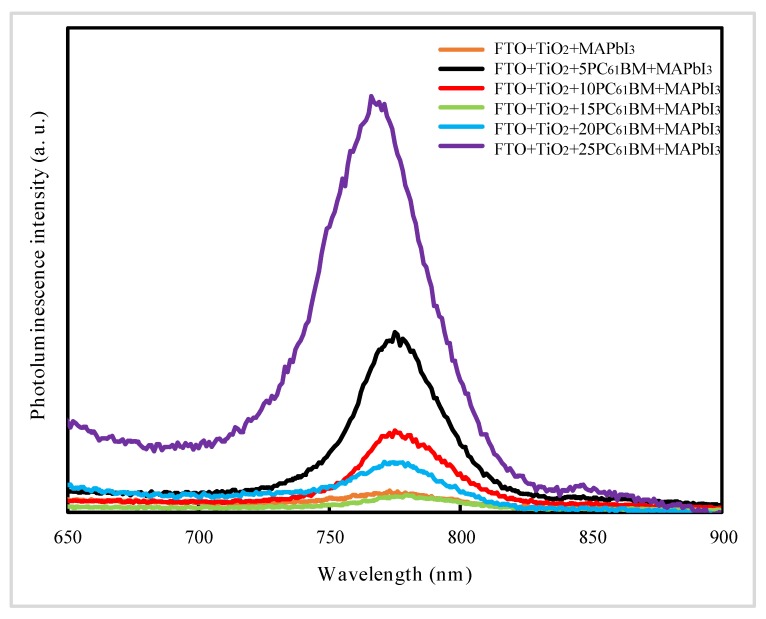
Photoluminescence spectra of perovskite films deposited on PC_61_BM of different concentration. The number 5, 10, 15, 20, 25 represent different concentration of PC_61_BM.

Finally, to investigate the influence of PC_61_BM substrate on the formation and properties of perovskite films, we studied the SEM images and absorbance spectra of perovskite films deposited on PC_61_BM of different concentration (5, 10, 15, 20, 25 mg/mL), absorbance spectra of PC_61_BM of different concentration and PL spectra of perovskite films deposited on PC_61_BM of different concentration, as shown in Figure 5, Figure 6 and Figure 7, respectively.

As shown in Figure 5, when the PC_61_BM precursor solution concentration is lower than 15 mg/mL, a good grained and uniform perovskite films were obtained. In contrast, obviously different perovskite surface morphologies are observed on 20 and 25 mg/mL PC_61_BM substrate, where the crystallinity of the perovskite films is distinctly deteriorated with serious grain boundary distortion and large holes.

From Figure 6a, we can observe that the absorption spectra signify the similar light-harvesting capabilities over 400 to 900 nm regardless of the different PC_61_BM concentration adopted. All five films exhibit approximately the same absorption edge at about 790 nm with no red-shift or blue-shift, corresponding to optical bandgap of perovskite. Particularly, perovskite films deposited on 25 mg/mL PC_61_BM manifest inferior light-harvesting capabilities, which is caused by the internal defects resulted from crystal distortion, and it is consistent with Figure 5j. From Figure 6b, we can observe that the PC_61_BM exhibits higher light-harvesting capabilities with the concentration increasing. However, perovskite films deposited on 15, 20 and 25 mg/mL PC_61_BM manifest similar absorbance, which indicates that light-harvesting capabilities of perovskite films deposited on PC_61_BM layers decrease with the precursor concentration (≥15 mg/mL) of PC_61_BM.

Figure 7 shows PL spectra of perovskite films deposited on PC_61_BM of different concentration. It is clearly observed that the peak of photoluminescence is fairly weak when nano TiO_2_ layer has not been optimized with PC_61_BM, being explained by the existence of via holes in nano TiO_2_ layer, which leads to leakage. Furthermore, from Figure 7, we can observe that the PL intensity decrease as the concentration of PC_61_BM increasing from 5 to 15 mg/mL. However, when the concentration of PC_61_BM is further increased to 25 mg/mL, the PL intensity raises up again and reaches the maximum value.

As demonstrated in a previous report, the PL intensity of the films is closely related to quenching of excitons resulted from the following two reasons, radiative relaxation of excited electrons back to the ground state of perovskite and electron injection from light-absorption layer into electron transport layer [24]. Hence, we can infer that the PC_61_BM of 15 mg/mL deposited on nano TiO_2_ layer guaranteed the high-quality interface between ETL and perovskite layer, resulting in more efficient injection of electrons from perovskite layer into nano TiO_2_ layer, leading to low PL intensity, which is consistent with Figure 5f.

**Figure 8:**
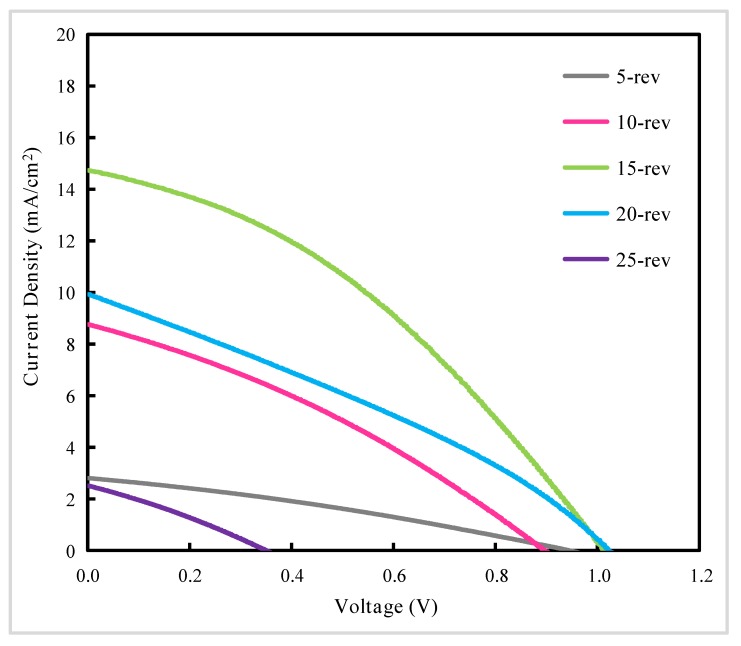
Reverse scanning J–V curves of perovskite solar cells optimized with different concentration of PC_61_BM. -rev represents reverse scanning. The number 5, 10, 15, 20, 25 represent different concentration of PC_61_BM. The device active area is 0.25 cm^2^ (0.5 cm × 0.5 cm).

We also have performed J–V characteristics of PSCs optimized with PC_61_BM, as shown in Figure 8 and Table 2. From Figure 8 and Table 2, we can discover that following the increasing of the concentration, the PCE extracted from reverse scanning J–V curves shows up a different trend about approximately 0.81%, 2.52%, 5.48%, 3.14% and 0.26% from the perovskite solar cells with PC_61_BM concentration of 5, 10, 15, 20 and 25 mg/mL respectively. It is clearly observed that the PCE reaches the maximum value when the concentration is increased up to 15 mg/mL, and then decreases with the further increasing of the concentration, which is consistent with the above discussion.

Finally, we demonstrate the carbon electrode-based flexible solar cell of PCE of 3.24% and 2.60% for reverse and forward scanning, respectively [25]. The carbon electrode is fabricated with spongy film composed of carbon nanoparticles, which leads to unstable contact in characterization, resulting in poor device performance. The golden electrode-based device is under investigation. Moreover, we are investigating the optimization process for perovskite layer and hole transport layer and the optimization process for the interface between layers to improve the device performance.

## 4. Conclusions

We have discovered a method to improve the performance of PSCs by optimizing low-temperature annealing process of solution-processed TiO_2_ ETL. In this work, we have received better nano TiO_2_ films using Process 1c (the as-grown nano TiO_2_ film had been delayed for one hour before the annealing temperature was raised from room temperature to 150 °C at rate of 8 °C/min). Moreover, the surface of nano TiO_2_ film optimized with 15 mg/mL PC_61_BM endows the device with better performance. Finally, we have demonstrated the carbon electrode-based flexible perovskite solar cell, and the golden electrode-based device is under investigation. Our results can serve as a platform to fabricate high-efficiency flexible PSCs and have a potential in flexible and wearable devices.

## Figures and Tables

**Table 1:** Data extracted from reverse scanning J–V curves of perovskite solar cells (PSCs) based on Process 1. The letter Di, Pr, De represent films prepared with Direct method, Proportional method, and Delayed method, respectively. The device active area is 0.25 cm^2^ (0.5 cm × 0.5 cm). The meaning of Process 1, Direct method, Proportional method and Delayed method is described in the second paragraph of experimental Section 2.2.2.

Samples	PCE ^a^ (%)	V_oc_^b^ (V)	J_sc_^c^ (mA/cm^2^)	FF ^d^
Di	0.26	0.55	1.94	0.27
Pr	0.11	0.47	0.93	0.25
De	0.96	0.66	4.82	0.30

Notes: a. PCE: power conversion efficiency; b. V_OC_: open-circuit voltage; c. J_SC_: short-circuit current; d. FF: fill factor.

**Table 2:** Data extracted from reverse scanning J–V curves of perovskite solar cells optimized with different concentration of PC_61_BM. -rev represents reverse scanning. The number 5, 10, 15, 20, 25 represent different concentration of PC_61_BM. The device active area is 0.25 cm^2^ (0.5 cm × 0.5 cm).

Samples	PCE ^a^ (%)	V_oc_^b^ (V)	J_sc_^c^ (mA/cm^2^)	FF ^d^
5-rev	0.81	0.94	2.81	0.31
10-rev	2.52	0.90	8.76	0.32
15-rev	5.48	1.01	14.74	0.37
20-rev	3.14	1.02	9.94	0.31
25-rev	0.26	0.35	2.52	0.29

Notes: a. PCE: power conversion efficiency; b. V_OC_: open-circuit voltage; c. J_SC_: short-circuit current; d. FF: fill factor.
